# A Case of Metastatic Parotiditis Terminating in Suppuration—Recovery

**Published:** 1887-06

**Authors:** T. M. Holmes

**Affiliations:** Rome, Ga.


### Clinic Reports.

### A CASE OF METASTATIC PAROTIDITIS TERMINATING IN SUPPURATION—RECOVERY.

BY T. M. HOLMES, M. D., ROME, GA.

Col. H., age fifty-years, a planter of the malarial belt of Middle Georgia, was on a visit to this city in August, 1885. I met him soon after his arrival and remarked the muddy and ichterous appearance of his skin. He felt badly, and, believing himself bilious, took several compound cathartic pills for the purpose of correcting this condition. These acted freely, but instead of relieving, very naturally aggravated his trouble. Thinking also that his kidneys were inactive, he undertook to relieve them by taking lager beer. He drank several glasses of this supposed diuretic, which was but adding fuel to the fire that had already torn to pieces the mucous membrane of the alimentary canal and hepatic ducts, producing an acute hepatitis and duodenitis with occlusion of the ductus communis choledochus. This, of course, prevented the bile from passing out through its proper exit, and being thus poured back upon the portal circulation, it was re-absorbed and transmitted to all parts of the system, which became thoroughly impregnated with this deleterious foreign substance. In consequence of the coloring ingredients of the bile, he was transformed into one of the darkest subjects I have ever seen.

Now, from the very nature of things, the kidneys were the only emunctories through which the bile could be eliminated, and in consequence of this, the urine became very dark. So prominent was this discoloration that I was at once apprehensive of its being a genuine case of malarial hæmaturia, with the inflammatory condition of the viscera referred to as a complication. A careful chemical and microscopical examination, however, dissipated this apprehension, as nothing more than bile and tube-casts could be discovered. Having now reached a diagnosis, I was more sanguine of his recovery, though an affection involving such extensive lesions was by no means to be accounted trivial. He was put upon phosphate of soda with the intent of relieving the inflammation of the affected parts. This treatment was persistently followed up for several days, but was not attended with the happy results for which I had hoped. It was also supplemented with diuretics for the purpose of exciting the kidneys to greater activity and thus eliminating the bile from the system. The noxious effects of this substance was being manifested in an alarming degree by various nervous phenomena, but the results of the treatment were now very satisfactory, as the complexion and urine gradually cleared up and inspired hopes of a speedy recovery. This proved, however, to be only illusory, as his temperature began to rise and soon reached 103°.

He complained of great tenderness at the angle of the jaw, on the left side. A careful examination was made, and the result was the discovery of an enlarging parotid gland. This issue was met by frequent applications of flax-meal poultices, with the hope of promoting resolution; but despite this, the gland rapidly enlarged, his temperature reached 105°, and the nervous phenomena increased in proportion. His symptoms were now so distressing that consultation was had, which resulted in giving five grains of quinine by the bowels every three hours and turpentine emulsion by the mouth. By this means his temperature was reduced. His mouth, which was very dry on account of the cutting off of the usual supply of saliva by the diseased gland, was rinsed frequently with pepper and vinegar, which had the very happy effect of exciting the salivary glands to greater activity. This course of treatment was persisted in until his nervous system seemed to be giving way under the heroic doses of quinine, when they were discontinued. By this time the gland had so enlarged as to close the left eye and to greatly disfigure the face. The muscles of that side of the face were exceedingly hard and swollen.

About the beginning of the third week of his illness, the gland suppurated and discharged very copiously through the external auditory meatus, while numerous particles of the gland substance, to the size of a pea, also passed off in this way. In a few days an exit formed just below the angle of the jaw, and through these two openings, it is no exaggeration to say that pus to the amount of from four to six quarts passed off during the three weeks that the process of suppuration was going on. This seems incredible, but can be attested by those faithful and numerous witnesses, composed of his wife, children and grand-children, who sat anxiously by his bedside, never leaving him night nor day. It really seemed that the process of suppuration and discharge would never cease, nor did it until the entire gland had passed away, leaving a cavity of two or three inches in circumference.

The discharge was continually encouraged by flax-meal poultices, which were repeated every hour, keeping the parts warm and soft, while the cavity was syringed out with warm carbolized water every three hours. The patient was kept on sweet milk, extract of malt, brandy and elixir of beef, iron and wine. He regained his strength slowly but surely, so that within about two months from the beginning of his illness he was able to return to the malarial atmosphere that had so poisoned him and shattered his constitution. His system was now thoroughly renovated and he gradually took on new life. Within a few months he weighed more than ever before. He remembered but little of the severe ordeal through which he had passed, though by this ordeal his mind sustained no permanent impairment.

In this case of metastatic parotiditis there were two exciting causes: (1) malarial poisoning, which had already debilitated his system, thereby putting it in a dependent state; (2) the lesions and active inflammation in the alimentary canal and hepatic ducts, the result of the pills. In this the glands of the bowels doubtless shared proportionately, thus making an affection similating enteric fever. From these lesions pus may have been transmitted to the parotid gland, which, with the poisonous effects of the bile and the high temperature, induced congestion, which resulted in a phlegmanous inflammation, changing the character of the saliva, and producing occlusion of the salivary ducts. These latter symptoms were evinced by the fact that during the whole course of his illness, from the beginning of the parotiditis, there was a distressing dryness of the mouth, and nothing flowed from the gland but a viscid, opalescent, ropy fluid that clung tenaciously to Steno's duct, and was ejected from the mouth with much difficulty. Virchow gives us a very graphic description of this affection as reported by Quain. He says "a tough, filamentous, whitish substance is formed within the duct, which is speedily transformed into pus. This invades the lobules of the gland, softening and breaking them down until the whole interstitial and gland tissue is more or less destroyed. When examined post mortem, the gland appears as if studded with numerous suppurating islands. The phlegmanous inflammation spreads from its seat of origin in various directions, most frequently to the neighboring connective tissue, enveloping the muscles found in this situation, descending to the clavicle, not even sparing the periosteum and bones, and it has even been known to pass to the brain and its coverings."

It can be readily seen, therefore, that an affection of so destructive a nature, and involving parts in such close proximity to the brain, would naturally be fraught with much danger.

The prognosis of this affection seems to depend mainly on the climate and the stage of the primary disease from which it originates. It will be borne in mind that it is purely symptomatic, being inter-current with typhoid and other continued or eruptive fevers, and is therefore necessarily modified in a greater or less degree by the causes of the primary affection. It is always an unfortunate complication, especially when it terminates in suppuration, as it prolongs in an unusual degree the illness of the already prostrated and wasting patient. Trousseau claims that it is an affection from which he has almost never seen enteric or other fever patients recover. Upon this Claud Muirhead remarks, " That this is certainly not in accord with the experience of England. If it occurs during convalescence from a fever or other disease, the prognosis is much more favorable."

The experience of Trousseau with this affection has doubtless been much more varied than mine, but out of several cases of metastatic parotiditis, in my own practice, occurring at various stages of fevers with enteric lesions, I have never lost a case. My experience, however, has been entirely in a pure, bracing, mountain atmosphere. I question very much if these happy results could be had in a hot and malarial section. Indeed, I have been informed by my able colleague and consultant, Dr. G. W. Holmes, who was for thirty years an active practitioner in Southwest Georgia, that the affection always proved fatal in that section.

---

STATISTICS OF TYPHOID FEVER.—In an Amsterdam graduation thesis, by Dr. M. Niemeijer, on the Statistics of Typhoid Fever, out of fifty cases in which complete observations were recorded, prodromata occurred in twenty-five, rigors in sixteen, "cold shivering" in one, pain in the left side in seven, diarrhœa in forty-two, splenic enlargement in forty-eight, rash in forty-seven, ileo-cæcal gurgling in nineteen, and pain in the same region in twenty, bronchial catarrh in forty-three, albuminuria in twelve, in three of which cystitis followed. In sixty-three cases relapses occurred six times. With regard to complications, out of seventy-three cases, intestinal hæmorrhage occurred in five, peritonitis in three, perforation occurring in two of these. Other complications were: pharyngitis, one; acute follicular sore-throat, one; parotitis, one; epistaxis, three; laryngeal perichondritis, one; pulmonary infiltration, thirteen; pleurisy with effusion, three; thrombus of the crural vein, one; acute nephritis, one, in a somewhat doubtful case; nephrolithiasis, two; herpes labialis, three; cutaneous hæmorrhage, three; periostitis of the tibia, one; joint affections, three; meningitis, one; neuralgia of the sole, one; profuse perspiration, two; polyuria, one; mental disturbance, four; deafness, five; bleeding from the ear, one. The total number of patients on whom observations were made was one hundred and ninety-four. Of these, twenty, or 10.3 per cent., died, the male mortality being decidedly higher than that occurring amongst female patients—12.5 per cent., as compared with 6 per cent.—*British Medical Journal, May 7, 1887.*

